# A phase 1 dose-escalation study on the safety, tolerability and activity of liposomal curcumin (Lipocurc^™^) in patients with locally advanced or metastatic cancer

**DOI:** 10.1007/s00280-018-3654-0

**Published:** 2018-08-03

**Authors:** Richard Greil, Sigrun Greil-Ressler, Lukas Weiss, Charlotte Schönlieb, Teresa Magnes, Bianca Radl, Gordon T. Bolger, Brigitta Vcelar, Peter P. Sordillo

**Affiliations:** 1 IIIrd Medical Department, Paracelsus Medical University Salzburg, Müllner Hauptstraße 45, 5020 Salzburg, Austria; 2 Salzburg Cancer Research Institute, Center for Clinical Cancer and Immunology Trials (SCRI-CCCIT), Salzburg, Austria; 3 Cancer Cluster Salzburg, Salzburg, Austria; 4 Nucro-Technics, 2000 Ellesmere Road, Unit 16, Scarborough, ON Canada; 5 Polymun Scientific Immunbiologische Forschung GmbH, Klosterneuburg, Austria; 6 SignPath Pharma Inc., New York, NY USA

**Keywords:** Curcumin, Lipocurc, Phase I cancer trials, Colon carcinoma, Prostate carcinoma, Hemolysis

## Abstract

**Purpose:**

This study was conducted to investigate the safety and tolerability of increasing doses of liposomal curcumin in patients with metastatic cancer. Investigations of anti-tumor activity and of the pharmacokinetics of curcumin were secondary objectives.

**Methods:**

In this phase I, single-center, open-label study in patients with metastatic tumors, liposomal curcumin was administered as a weekly intravenous infusion for 8 weeks. Dose escalation was started at 100 mg/m^2^ over 8 h and the dose increased to 300 mg/m^2^ over 6 h.

**Results:**

32 patients were treated. No dose-limiting toxicity was observed in 26 patients at doses between 100 and 300 mg/m^2^ over 8 h. Of six patients receiving 300 mg/m^2^ over 6 h, one patient developed hemolysis, and three other patients experienced hemoglobin decreases > 2 g/dL without signs of hemolysis. Pharmacokinetic analyses revealed stable curcumin plasma concentrations during infusion followed by rapid declines to undetectable levels after the infusion. Anti-tumor activity by RECIST V1.1 was not detected. Significant tumor marker responses and transient clinical benefit were observed in two patients.

**Conclusion:**

300 mg/m^2^ liposomal curcumin over 6 h was the maximum tolerated dose in these heavily pretreated patients, and is the recommended starting dose for anti-cancer trials.

## Introduction

Curcumin is a natural product found in the plant turmeric, with the chemical name diferuloylmethane. Its molecular formula is C_21_H_20_O_6_ with a molar mass of 368.38 g/mol. Curcumin acts as an anticancer therapeutic by promoting death pathways and limiting survival pathways in tumor cells [1]. Despite its activity against mature cancer cells and cancer stem cells, curcumin is known to have little toxicity against normal cells, even with long-term exposure [2–5]. This may be because the uptake of curcumin is much greater in cancer cells than in normal cells [6, 7]. Curcumin also has disparate effects on cancer and normal stem cells. Through its anti-inflammatory effects, curcumin changes the microenvironment around the cancer cell to one that is adverse to proliferation of cancer stem cells but conducive to normal stem cells [8]. In fact, curcumin has been shown, in multiple studies, to have stimulatory and protective effects on normal stem cell function [9–15].

A limiting factor for the therapeutic use of curcumin is its poor water solubility and corresponding poor bioavailability after oral intake [16, 17]. To overcome the pharmacokinetic and bioavailability limitations of oral administration, a liposomal formulation of curcumin was developed for intravenous administration and represents a promising drug delivery system [18–23].

The anticancer activity of liposomal curcumin was demonstrated in xenograft models with pancreatic cell lines BxPC-3 or MiaPaCa-2 [18–20, 22]. The pharmacokinetics and the organ and tissue distribution of curcumin and its active metabolite tetrahydrocurcumin (THC) were investigated in beagle dogs after 2 and 8 h continuous infusions of liposomal curcumin. While the 2-h infusions led to higher plasma concentrations of curcumin and THC than the 8-h infusions [21], tissue and organ concentrations of curcumin and THC were generally higher after the 8-h infusions [24].

Liposomal curcumin was tested in a randomized, placebo-controlled, double-blind, dose-escalation study in 49 healthy male and female volunteers. Subjects received a single dose of liposomal curcumin (10–400 mg/m^2^; *n* = 2–6 per group, total *n* = 39) or placebo (*n* = 10) over 2 h intravenously. Liposomal curcumin was well tolerated, but a transient red blood cell echinocyte formation, known to occur with both liposomes and with curcumin, with a concomitant increase in mean cellular volume, was observed at dosages ≥ 120 mg/m^2^ [23].

The primary objective of the current study was to evaluate the safety and tolerability of liposomal curcumin administered as an intravenous infusion in patients with locally advanced or metastatic cancer. Secondary objectives were to determine a recommended starting dose for phase II studies, to evaluate the pharmacokinetics (PK) of curcumin and THC during and following infusion #1 and to evaluate the antitumor activity of liposomal curcumin according to RECIST V1.1 {proportion of patients reaching complete response (CR)/[partial response (PR)/stable disease (SD) after 8 weeks]}.

## Materials and methods

### Study drug

Curcumin, (1E6E)-1,7-bis (4-hydroxy-3-methoxyphenyl)-1,6-heptadiene-3,5-dione, molecular weight 368.38 g/mol, was synthesized at Sami Labs Limited (Bangalore, India) under Good Manufacturing Practice (GMP) with a purity of 99.2%. Liposomal curcumin was then manufactured, tested, packaged and labeled by Polymun Scientific, Austria, in compliance with GMP as described in a previous study [23]. Liposomal curcumin was provided in 20-mL glass vials containing 20 mL liposomal suspension, with a curcumin concentration of 6.0 ± 1.5 mg/mL.

### Patients

Male and female patients ≥ 18 years with a histologically/cytologically confirmed diagnosis of locally advanced or metastatic cancer, for whom no anti-tumor therapy of proven benefit was available at study enrolment, were eligible for this study. Key inclusion criteria were ECOG performance status 0–2, life expectancy of at least 3 months and the presence of at least one measurable lesion by RECIST v1.1. Hematological inclusion criteria included, absolute neutrophil count ≥ 1500 cells/µL, hemoglobin (Hb) > 9.5 g/dL and a platelet count > 100,000 µL. Inclusion criteria based on renal function included > 50 mL/min with estimated creatinine clearance (eCcr) using the Cockcroft–Gault formula or serum creatinine < 1.5 mg/dL. Clinical chemistry inclusion criteria included, total serum bilirubin < 3.0 mg/dL, and AST and ALT less than five times the upper limit of normal (ULN). Each patient had to provide a signed informed consent. Exclusion from study criteria included patients with lymphoma, other hematological cancers or glioblastoma multiforme. Other key exclusion criteria were active infection, a fever > 38.5 °C within 3 days prior to the first day of study drug dosing and evidence of disease (hemolytic diathesis, hemochromatosis) that could be exacerbated by administering liposomal curcumin. The concurrent use of any medications classified as cytochrome p450 inhibitors or inducers, systemic therapy less than 3 weeks before the day of first study treatment and unresolved toxicities from prior systemic anti-cancer therapy except symptomatic motor or sensory neuro-toxicities NCI-CTC Grade ≤ 2 were also criteria for exclusion. Cardiovascular study exclusion parameters included clinically significant ECG aberrations according to the discretion of the investigator, left ventricular ejection fraction (LVEF) < 50%, NYHA Class 2 or congestive heart failure, uncontrolled hypertension and cardiac arrhythmias. Known positive HIV serology or evidence of active hepatitis and women who were pregnant, breast feeding, and not taking contraceptive measures were also criteria for exclusion from study participation.

The study was conducted at the IIIrd Medical Department of the Paracelsus Medical University Salzburg and the Salzburg Cancer Research Institute. The study protocol was approved by the Ethics Committee of the province Salzburg [Clinicaltrials.gov identifier NCT02138955, European Clinical Trials Database (EudraCT) number 2013-001594-24]. The study was conducted in accordance with the Declaration of Helsinki, Good Clinical Practice guidelines, and all local and federal regulations. All patients provided written informed consent.

### Study design and dose escalation

This was an open-label, single-center, dose-escalation study. Study medication was given once per week (± 1 day). In order to reduce the chance of allergic reactions, patients were pre-medicated with 50 mg diphenhydramine before each treatment. Individual patients received treatment either on study until the completion of eight cycles (8 weeks), until tumor progression or until intolerable toxicity was observed. If on week 8, the patients exhibited objective clinical benefit, they could be offered the option of additional liposomal curcumin at the same dose and schedule they previously received.

The starting dose of 120 mg/m^2^ over 24 h was based on the results in the phase 1a safety study in healthy subjects. After the second infusion in the first patient, the study was temporarily interrupted due to precipitate formation in the infusion line. The patient did not experience any adverse events related to this incident. After extensive in-use stability tests, a substantial amendment was filed with regulatory authorities and the concerned EC, and the study was restarted in the first cohort of patients with infusions of 8 h duration at a dose level (DL) of 100 mg/m^2^ liposomal curcumin. Dose escalation to the next DL was permitted if no dose-limiting toxicity (DLT) occurred during the first 3 weeks in three evaluable patients and the Data Safety Monitoring Board (DSMB) recommended dose escalation. For patents 5–27, escalating liposomal curcumin doses were given as 8 h infusions to a maximum of 300 mg/m^2^ (Table 1). The final six patients (patients 27–32) also received a dose of 300 mg/m^2^, but the infusion time in these patients was shortened to 6 h. Planned escalations beyond this dose were not done because of the possible development of hemolytic toxicity secondary to curcumin at this dose level.

**Table 1:** Patient characteristics

Curcumin DLs	Other	DL 1	DL 2	DL 3	DL 4	DL 5	DL 6	DL 6a	All
(mg/m^2^)	120	100	120	150	190	240	300	300	**–**
Duration of infusion									
(h)	24	8	8	8	8	8	8	6	–
Number of patients									
* n*	1	4	7	4	3	3	4	6	32
Gender									
Male									
*n* (%)	1	4 (100)	5 (71)	2 (50)	1 (33)	2 (67)	4 (100)	4 (67)	23 (72)
Female									
*n* (%)	0	0 (0)	2 (29)	2 (50)	2 (67)	1 (33)	0 (0)	2 (33)	9 (28)
Age (years)									
Median	65.2	63.7	58.2	72.8	61.8	60.6	67.0	60.3	62.7
Range	65.2	50.4–68.9	42.6–69.8	66.7–84.5	48.2–62.2	58.6–69.4	57.0–76.9	48.0–72.9	42.6–84.5
Number of previous treatment regimes									
2									
*n*	1	**–**	**–**	**–**	**–**	**–**	**–**	**–**	1
3									
*n*	**–**	**–**	1	1	1	**–**	**–**	**–**	3
4									
*n*	**–**	2	1	4	**–**	1	1	1	10
5									
*n*	**–**	1	5	5	**–**	**–**	2	1	14
6									
*n*	**–**	1	2	**–**	1	**–**	**–**	**–**	4
7									
*n*	**–**	**–**	1	**–**	**–**	**–**	1	1	3
8									
*n*	**–**	**–**	**–**	**–**	1	1	**–**	2	4
9									
*n*	**–**	**–**	**–**	**–**	**–**	**–**	**–**	1	1
10									
*n*	**–**	**–**	**–**	**–**	**–**	1	**–**	**–**	1
ECOG score									
0									
*n*	1	**–**	2	1	1	1	**–**	3	9
1									
*n*	**–**	4	3	3	1	1	2	2	16
2									
*n*	**–**	**–**	2	**–**	1	1	2	1	7
Mean treatment duration									
(days)	15	31	33.3	27.5	38.3	38.3	26.8	54.2	35.8

### Safety assessments

Safety assessments consisted of the monitoring and recording of all adverse events (AEs) and serious adverse events (SAEs), the regular monitoring of blood counts and blood chemistries, and regular physical examinations and measurements of vital signs. DLTs were defined as described below.

Hemolysis (NCI-CTC grade 2: evidence of hemolysis and ≥ 2 g decrease in Hb in two consecutive measurements within 120 min after the end of infusion, confirmation of a causal relationship to the study medication according to the investigator); NCI-CTC Grade 3 or 4 toxicities (excluding nausea and vomiting, which responds to antiemetic treatment and alopecia); prolonged (> 2 weeks) NCI-CTC Grade 2 toxicities (neuro-cerebellar: intention tremor, slurred speech, nystagmus, dysmetria); NCI-CTC Grade 4 platelet toxicities; NCI-CTC Grade 4 granulocyte toxicity ≥ 7 days; and febrile neutropenia: defined as an absolute neutrophils count < 500/mm^3^ and fever either as two elevations of oral temperature > 38 °C with 1 h interval or a single oral temperature, > 38.5 °C, provided that this single episode is not clearly related to other events.

### Pharmacokinetic assessments

Blood samples were collected into K_3_-EDTA containing tubes for the determination of free curcumin and THC plasma concentrations by liquid chromatographic–tandem mass spectrometry, using a validated method at Nucro Technics (Canada), as previously reported for a study with liposomal curcumin [23]. Samples for infusion #1 were collected at baseline (BL), 2 h (2 h) after start of infusion, and after end of infusion (EOI) = 0, 10, 20, 30, 45, 60 and 120 min and at EOI for infusions #2, #3, #5 and #8. A preliminary PK analysis of the samples obtained from patients treated in dose level 2 that was requested by the safety data monitoring board revealed that curcumin plasma levels were only detectable during infusion (at 2 h) but not after the end of infusion. To allow better characterization of the pharmacokinetics of curcumin in plasma the samples collection time points were adapted (Amendment 3, starting from Patient #17) to BL, 2, 4 and 6 h during infusion and EOI, 10, 20, 30 and 45 min after EOI. The number of blood collections remained unchanged.

The PK analysis focused on patients participating in the study after amendment 3 who had curcumin plasma concentration values above the limit of quantitation (LOQ = 10 ng/mL). These patients had received 150, 190, 240, or 300 mg/m^2^ doses of curcumin over 8 h of infusion or received 300 mg/m^2^ over 6 h of infusion. Due to a lack of concentration data following infusion for the majority of patients, the pharmacokinetic parameters determined were *C*_max_, *T*_max_, AUC_0−Tlast_ and *C*_last,_ using validated Phoenix WinNonlin Professional Software (v6.3).

Dose proportionality and linearity was assessed using the plasma concentrations of curcumin determined at 2 h during infusion compared to the rate of infusion and linear regression of the plot of infusion rate versus the 2 h plasma concentration of curcumin. The rate of infusion was used instead of the dose of curcumin to compare data from infusions of different duration.

### Efficacy assessments

Efficacy endpoints were tumor response rate (complete response/partial response/stable disease/progressive disease) according to RECIST v1.1 after 8 weeks, changes in serum cancer markers that are normally assessed in the management of the patient’s cancers, and changes in clinical status.

## Results

### Patients

32 patients with metastatic cancer were treated with liposomal curcumin infusions at doses between 100 and 300 mg/m^2^ and infused over either 8 h (dose levels 100–300 mg/m^2^), 6 h (dose level 300 mg/m^2^) or in one patient 24 h (dose level 120 mg/m^2^) (Table 1). The median age of patients at study entry was 62.7 years (range 42.6–84.5 years) and the majority were male (71.9%). The primary cancer diagnoses and number of patients diagnosed are were uveal melanoma—1; squamous carcinoma, unknown primary—1; squamous carcinoma of head and neck—2; carcinoma of the parotid gland—1; breast carcinoma—1; lung carcinoma—1; squamous carcinoma, esophagus—2; adenocarcinoma, esophagus or stomach—5; hepatocellular carcinoma—2; cholangiocarcinoma—3; adenocarcinoma of pancreas—3; adenocarcinoma, small bowel—1; colon carcinoma—1; anal epidermoid carcinoma—2; urothelial carcinoma—3; uterine carcinoma—1; prostate carcinoma—2. Patients were heavily pretreated with a median of five previous lines of therapy (range 2–10) (Table 1). The patients received between one and 11 infusions of liposomal curcumin, and the median treatment duration was 38.5 days (range 1–82 days) (Table 1). 17 patients (53.1%) received at least five infusions of the study medication, whereas eight patients (25.0%) received all eight planned infusions. Treatment was stopped after disease progression (23 patients), deterioration of general medical condition (six patients), early withdrawal (two patients) or study interruption (one patient). Ten patients died within 4 weeks after the last study treatment.

### Adverse events (AEs)

A total of 143 AEs were experienced by 30 patients (93.8%). Of these AEs, only 34 AEs (in 14 patients) were considered definitely, probably or possibly related to the study treatment, while the others were considered related to underlying disease. Infusions of liposomal curcumin over 8 h at DL 1–6 (100–300 mg/m^2^) were generally well tolerated. At DL 6a (300 mg/m^2^ over 6 h), the number of observed hematological AEs considered related to the study drug increased notably (Table 2).

**Table 2:** Adverse events with definite or probable causal relationship to study treatment

DL	Patient	AE (preferred term)	Timing	Grade	SAE	Relationship	Outcome
1	002	Red blood cell abnormality	Cycle 1	n.a.	No	Definite	Recovered
	003	Platelet count decreased	Cycle 1	2	No	Probable	Recovered with sequelae
		Platelet count decreased	Cycle 2	2	No	Probable	Recovered with sequelae
	004	Pyrexia	Cycle 1	1	No	Probable	Recovered
		Productive cough	Cycle 1	1	No	Probable	Recovered
		Pyrexia	Cycle 2	1	No	Probable	Recovered
		Chills	Cycle 5	2	No	Probable	Recovered
		Pyrexia	Cycle 5	1	No	Probable	Recovered
5	023	Hypertrichosis	Cycle 4	1	No	Probable	Recovered
6a	028	Anemia	Cycle 3	2	No	Probable	Ongoing after final examination
	028	Haemolysis	Cycle 4	3	No	Definite	Recovered with sequelae
	029	Anemia	Cycle 4	3	No	Definite	Ongoing after final examination
	030	Infusion-related reaction	Cycle 7	2	No	Probable	Recovered
	031	Face edema	Cycle 1	2	Yes	Probable	Recovered
	032	Anemia	Cycle 1	3	Yes	Probable	Recovered
	032	Anemia	Cycle 2	2	No	Definite	Recovered

Of the 40 reported serious adverse events (SAEs) in 23 patients (71.9%), two were related to study treatment (Patient #31 facial edema; Patient #32 anemia). Echinocytes were observed in one patient (#2) in DL 1 (100 mg/m^2^). Cardiac, pulmonary, hepatic or renal toxicity related to the study medication was not observed. Only three Grade 1 gastrointestinal AEs, possibly related to study medications, were observed.

A total of four AEs were rated Grade 3 by the investigator (anemia in patients #29 and #32; hemolysis in Patient #28; hyponatremia in Patient #3). Hemolysis and anemia were observed at DL 6a and resulted in discontinuation of dose escalation (see DLT). Patient #3, with a history of liver cirrhosis, ascites, generalized edema and a pre-existing hyponatremia, developed a Grade 3 hyponatremia with a decrease in serum sodium from 133 mmol/L before the first infusion to a low of 126 mmol/L before the sixth infusion.

### DLT and recommended phase 2 dose

Six patients were included in DL 6a. One patient (#28) displayed definite signs of hemolysis. During cycle 2, the patient’s Hb decreased from 11.5 g/dL (start of infusion) to 9.5 g/dL (EOI) to 9.1 g/dL (3 h after EOI) and haptoglobin decreased slightly. During cycle 4, Hb decreased from 8 g/dL (start) to 6.4 g/dL (EOI) to 5.5 g/dL (2.5 h after EOI) and haptoglobin decreased considerably from 130 mg/dL (start) to 89 mg/dL (EOI) to 15 mg/dL (2.5 h after EOI) and then to < 10 mg/dL (15 h after EOI) (normal haptoglobin range 30–200 mg/dL). In both treatment cycles, this patient also showed elevated bilirubin, percent reticulocytes and LDH. Hemolysis related to study treatment was documented as adverse event for cycle 4. Between cycles 1 and 2, the patient suffered from port-a-cath infection (SAE) and received an erythrocyte transfusion. Between cycles 3 and 4, the patient had anemia grade 2, probably related to a concurrent infection, and received another erythrocyte transfusion. Blood smears showed no fragmentocytes or echinocytes.

A clinically significant drop of Hb (> 2 g/dL) related to infusion of liposomal curcumin was also observed in three other patients (#29, #32, #33), however the presence hemolysis was not definite in these patients since haptoglobin and MCV did not change. Blood smears showed no fragmentocytes or echinocytes. In addition, the patients showed no signs of bleeding. In Patient #32, Hb decreased to 5.5 g/dL after cycle 2 and the observed anemia was documented as a serious adverse event (SAE) with definite relationship to study treatment (SUSAR). Patients #30 and #31 did not experience clinically significant drops in Hb.

One patient (#31) developed a facial edema (Grade 2) during the night after his first infusion, which was treated with dexamethasone and dibondrin, and required a prolongation of hospitalization for further observation. The investigator suspected a causal relationship between the facial swelling and the administration of curcumin. The patient recovered within 1 day and study treatment was continued without further occurrence of facial edema.

The DSMB unanimously decided not to include any more patients into a higher dose level (DL 7), but instead to stop enrolment into the study. No further patients were to be enrolled into the previous dose level (DL 6), since one definite DLT was observed in DL 6a. The current dose level was not expanded to nine patients since no further gain in information was expected.

### Pharmacokinetics of curcumin and THC

**Fig. 1:**
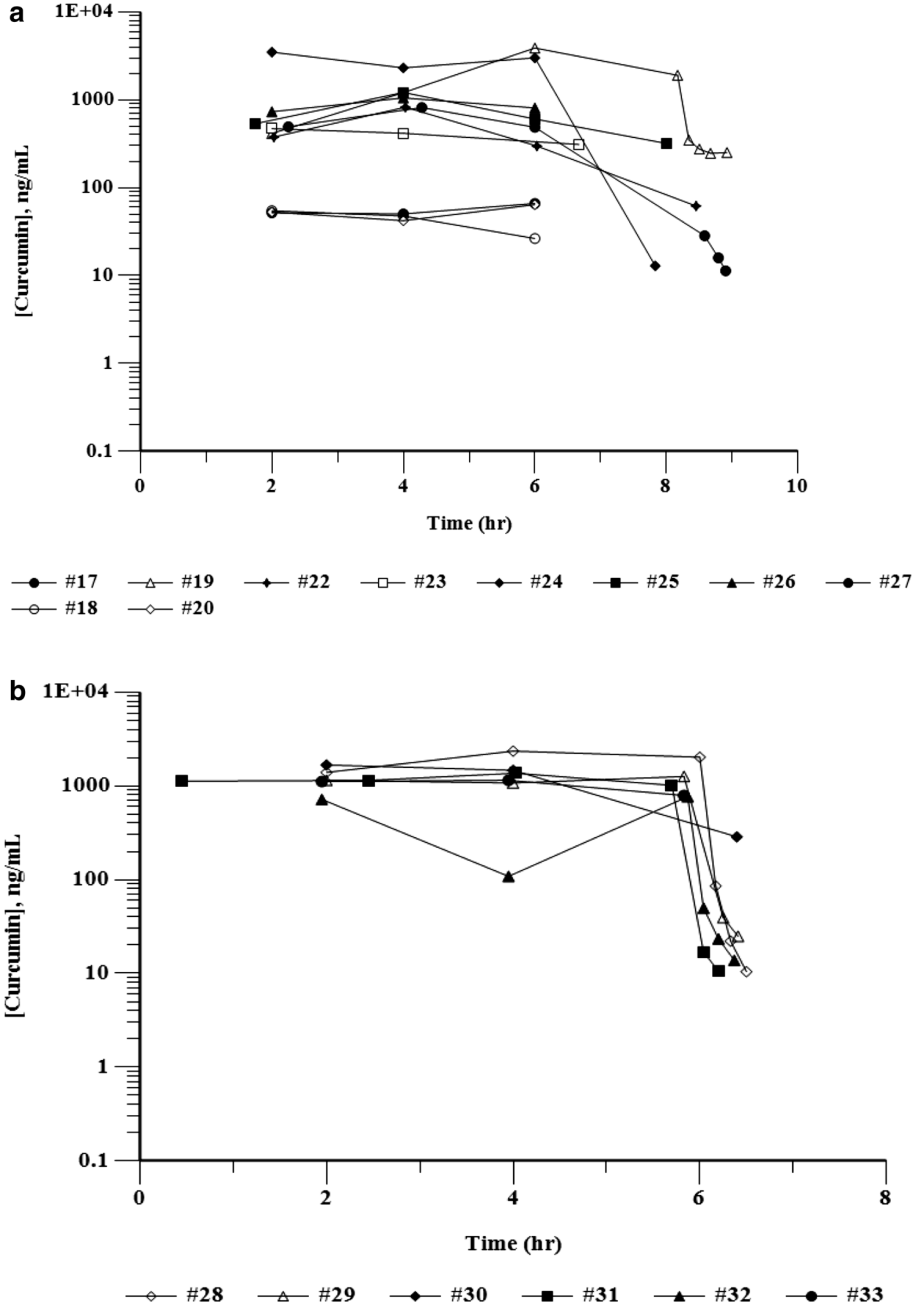
Curcumin plasma concentration curves during infusion with curcumin. Plasma levels of curcumin are shown for individual subjects. Time (h) represents the actual sampling times. Time “0” represents the start of infusion. **a** Patients #17–#27 receiving liposomal curcumin over 8 h. **b** Patients #28–#33 receiving liposomal curcumin over 6 h

**Fig. 2:**
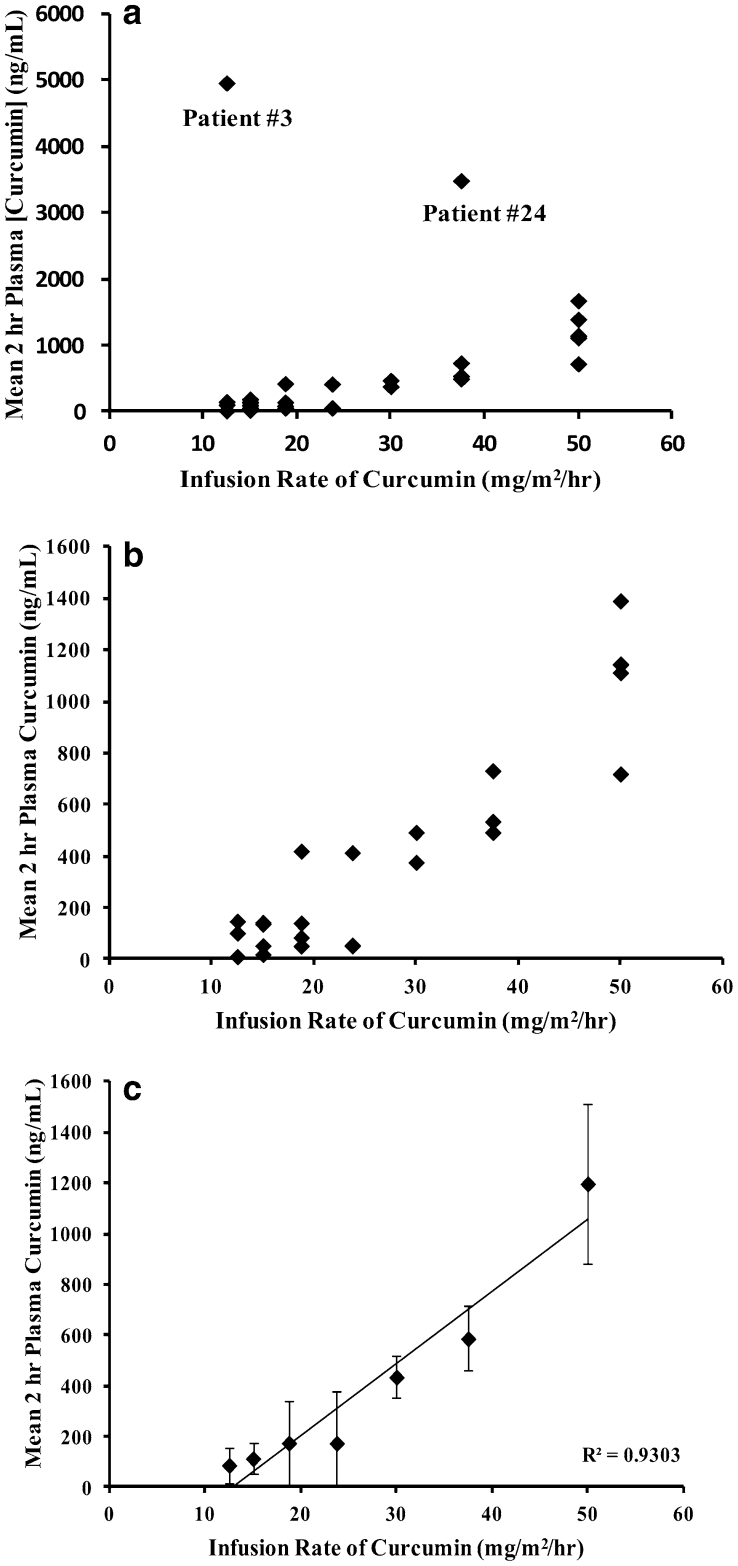
Plasma levels of curcumin at 2 h during infusion compared to the infusion rate. **a** For all patients with exception to patients #21 because of interruption of infusion, **b** for patients with the exclusion of patients #3, #21 and #24, **c** mean ± SD at each infusion rate for data shown in **b**. The infusion rate normalized 2 h curcumin levels were 7.0, 7.6, 9.3, 7.3, 14.5, 15.6, and 24.0 and infusion rates of 12.5, 15.0, 18.75, 23.75, 30.0, 37.5 and 50 mg/m^2^/h, respectively

Blood samples for the PK evaluation of total curcumin (plasma protein bound and free) and total THC were collected and the plasma isolated from all patients who received the first infusion of liposomal curcumin over 8 or 6 h. Patient #21 was excluded from the PK analysis because the infusion was interrupted for 1.5 h. For patients treated at dose levels DL 1–DL 3 (100–150 mg/m^2^) who had only one blood collection during infusion (before amendment 3), the PK parameters were not evaluated since most of the plasma samples were below the limit of quantification (BLOQ) for curcumin and THC. PK parameters for patients #17–#33 are presented in Table 3. Individual curcumin plasma concentrations–time curves for these patients are depicted in Fig. 1. The mean *T*_max_ for curcumin was achieved during infusion, and ranged from 3.6 to 4.0 h across a dose range of 190–300 mg/m^2^ for the 8-h infusion and 4.2 h for the 6-h infusion. While steady-state levels of curcumin were achieved for most of the patients, Patient #19 did not achieve steady-state levels of curcumin with plasma levels increasing between 2 and 6 h of infusion from 413.81 to 3885.14 ng/mL (Fig. 1), resulting in a considerably higher AUC_0−Tlast_ value compared to other patients in the 190 mg/m^2^ dose group (Table 3). In general, however, AUC_0−Tlast_ values increased with increasing infusion doses of curcumin. At the EOI, plasma levels rapidly decreased to BLOQ within 10 min. In order to compare the plasma levels of curcumin achieved after different infusion doses and infusion times and to include as many data points as possible, the plasma levels of curcumin in patients following 2 h of infusion were compared to the infusion rates. Figure 2a depicts the relationship between infusion rate and curcumin plasma levels of patients at 2 h during infusion with dose levels of 100, 120, 150, 190, 240 and 300 mg/m^2^ over 8 h and 300 mg/m^2^ over 6 h, corresponding to infusion rates of 12.5, 15, 18.75, 23.75, 37.5 and 50 mg/m^2^/h, respectively. It is apparent that patients #3 and #24 had very high 2 h plasma concentrations compared to all other patients. In order to understand the relationship between infusion rate and plasma concentrations for the remaining patients, the data for patients #3 and #24 were removed (Fig. 2b). Mean plasma concentrations of curcumin for the remaining 29 patients showed an apparent linear dependence on infusion rate (*R*^2^ = 0.9303). However, infusion rate normalized mean plasma concentrations of curcumin ranged between 7.0 and 9.3 up to an infusion rate of 23.75 mg/m^2^/h and ranged between 14.5 and 24.0 between infusion rates of 30–50 mg/m^2^/h, suggesting greater than dose proportional increases of plasma curcumin concentrations at higher infusion rates (Fig. 2c).

**Table 3:** PK parameters for curcumin

Patient	*C*_max_ (ng/mL)	*T*_max_ (h)	AUC_0−Tlast_ (ng h/mL)	*C*_last_ (ng/mL)	*T*_last_ (h)
DL 3: 150 mg/m^2^, 8 h infusion					
17	66.1	6.0	270	66.1	6.0
DL 4: 190 mg/m^2^, 8 h infusion					
18	54.7	2.0	231	26.4	6.0
19^a^	3885.1	6.0	13,752	251.2	8.8
20	64.1	6.0	254	64.1	6.0
Mean	59.4	4.0	243	45.3	6.0
SD	6.7	2.8	16	26.7	0
SE	4.7	2.0	12	18.8	0
%CV	11	71	7	59	0
DL 5: 240 mg/m^2^ 8 h infusion					
22	824.2	4.0	3077	61.9	8.5
23	469.4	2.0	2325	310.4	6.7
Mean	646.8	3.0	2701	186.2	7.6
SD	250.9	1.4	532	175.7	1.3
SE	171.4	1.0	376	124.3	0.9
%CV	39	48	20	94	17
DL 6: 300 mg/m^2^, 8 h infusion					
24	3484.4	2.0	17,351	12.9	7.8
25	1212.3	4.0	5174	319.1	8.0
26	1051.4	4.0	4374	807.1	6.0
27	815.8	4.3	3674	11.3	8.9
Mean	1641	3.6	7643	287.6	7.7
SD	1239.7	1.1	6501	375.4	1.2
SE	715.7	0.6	3753	216.7	0.7
%CV	76	30	85	131	16
DL 6a: 300 mg/m^2^, 6 h infusion					
28	2351.2	4.0	9701	10.4	6.5
29	1261.8	5.8	5780	24.9	6.4
30	1672.7	2.0	6915	286.2	6.4
31	1367.6	4.0	6669	10.6	6.2
32	767.2	5.9	2448	13.8	6.4
33	1147.7	4.0	5168	790.2	5.8
Mean	1428	4.3	6113	189.4	6.3
SD	540.1	1.4	2378	313.8	0.2
SE	220.5	0.6	971	128.1	0.1
%CV	38	34	39	166	4

^a^Data for Patient #19 were not included in the calculation of the mean, SD, SE and %CV

The pattern of plasma concentrations for THC was similar to that for curcumin, but the plasma levels of THC expressed as percentage of the AUC_0−TLast_ of THC to curcumin were considerably lower ranging from 2.1 to 21.8% (mean of 8.5%) of the plasma levels of curcumin at 2–4 h across individual patients during infusion. The PK parameters for THC are shown in Table 4 (see supplementary materials).

**Table 4:** PK parameters for THC

Patient	*C*_max_ (ng/mL)	*T*_max_ (h)	AUC_0−Tlast_ (ng h/mL)	*C*_last_ (ng/mL)	*T*_last_ (h)	AUC_0−Tlast_ THC/AUC_0−Tlast_ curcumin
DL 3: 150 mg/m^2^, 8 h infusion						
17	16.1	4	59	10.3	6.0	0.218
DL 4: 190 mg/m^2^, 8 h infusion						
18	7.7	4.0	22	7.7	4.0	0.096
19^a^	396.1	8.1	1656	11.8	8.8	0.122
20	6.6	6.0	31	6.6	6.0	0.121
Mean	7.2	5.0	27	7.2	5.0	0.109
SD	0.7	1.4	6	0.7	1.4	0.018
SE	0.5	1.0	4	0.5	1.0	0.012
%CV	10	28	23	10	28	16
DL 5: 240 mg/m^2^ 8 h infusion						
22	24.3	2.0	102	14.3	6.0	0.033
23	63.3	2.0	271	28.2	6.7	0.116
Mean	43.8	2.0	186	21.2	6.4	0.075
SD	27.6	0	119	9.9	0.5	0.059
SE	19.5	0	84	7.8	0.3	0.042
%CV	63	0	64	47	7	79
DL 6: 300 mg/m^2^, 8 h infusion						
24	108.3	2.0	359	48.2	6.0	0.021
25	75.2	3.8	407	28.6	7.6	0.079
26	75.6	2.0	426	5.3	8.5	0.097
27	38.5	2.3	187	5.1	8.6	0.051
Mean	74.4	2.5	344	21.8	7.7	0.062
SD	28.5	0.8	109	20.8	1.2	0.033
SE	16.5	0.5	63	12.0	0.7	0.019
%CV	38	34	32	95	16	54
DL 6a: 300 mg/m^2^, 6 h infusion						
28	110.4	2.0	497	6.9	6.8	0.051
29	35.1	4.0	155	5.2	6.8	0.027
30	80.1	2.0	385	5.2	7.3	0.056
31	67.6	2.5	332	6.0	6.0	0.050
32	70.7	5.9	321	8.4	6.6	0.131
33	58.3	5.8	264	5.7	6.2	0.051
Mean	70.3	3.7	326	6.2	6.6	0.061
SD	24.9	1.8	115	1.2	0.5	0.036
SE	10.2	0.7	47	0.5	0.2	0.015
%CV	35	49	35	20	7	59

^a^Data for Patient #19 were not included in the calculation of the mean, SD, SE and %CV

### Efficacy

The primary efficacy endpoint was the response rate (complete response/partial response/stable disease/progressive disease) according to RECIST v1.1 after 8 weeks. 23 patients had a tumor response assessment but only eight patients reached the tumor assessment after 8 weeks of treatment. All of them showed progressive disease (PD). Of 15 patients with tumor assessment between weeks 4 and 8, 14 showed PD and one (Patient #27) showed stable disease (SD). Five patients experienced a deterioration of their general condition after 1–4 drug infusions and treatment was stopped without tumor assessment. Two patients withdrew their informed consent.

**Fig. 3:**
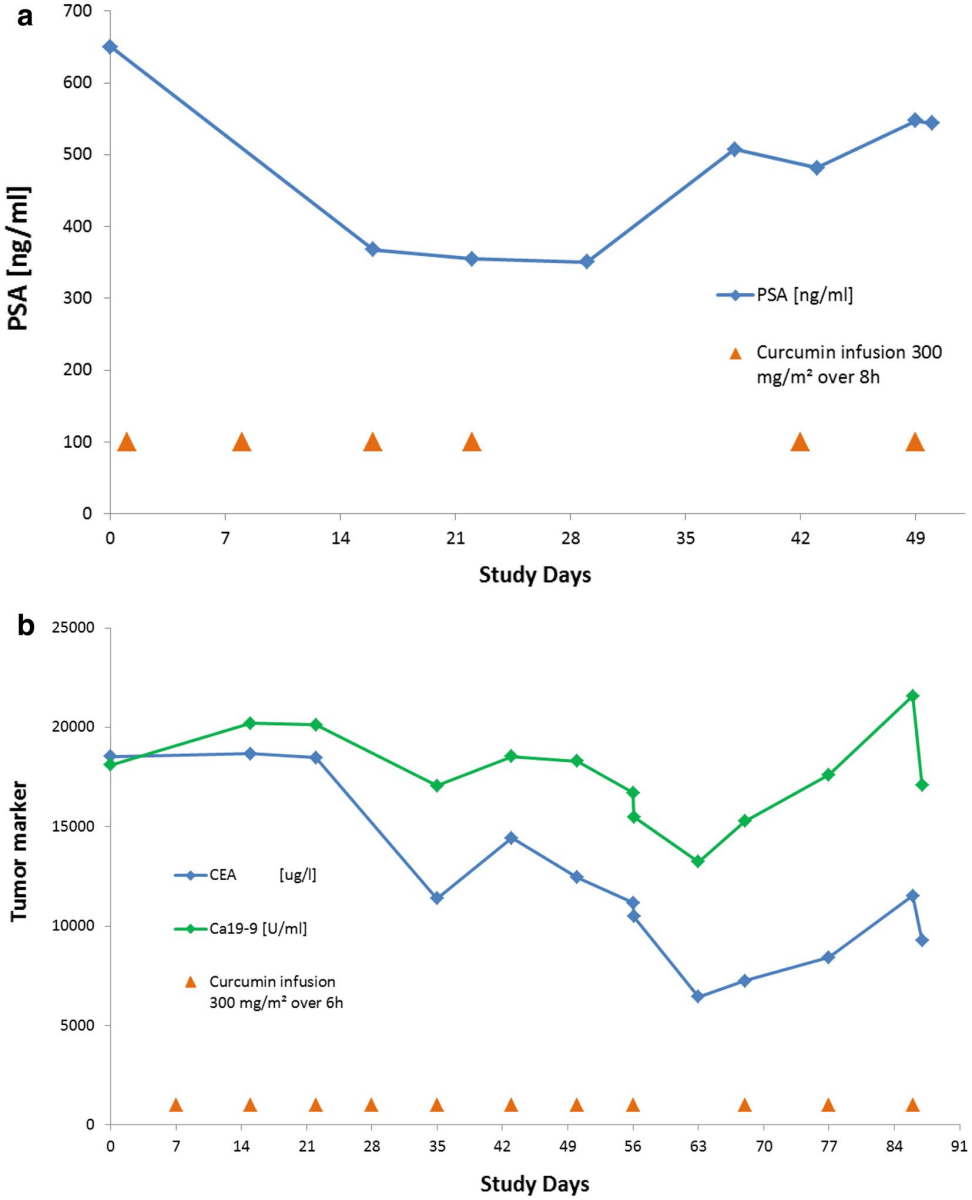
Time course of tumor marker. **a** Time course of PSA (ng/mL) in Patient #27; **b** time course of CEA (μg/L) and Ca19-9 (U/mL) in Patient #30

Tumor markers CEA, CA19-9, PSA and CA15-3 were assessed where relevant. In two patients, a significant but temporary reduction of a tumor marker was observed. In Patient #27 with prostate cancer and bone and lymph node metastases as well as lymphangiosis of the lung, the PSA level was reduced from 649 to 355 ng/mL after the fourth infusion. After the fourth infusion treatment was interrupted for 3 weeks due to a suspected lung infection, the PSA level increased again to 547 ng/mL (Fig. 3a). A tumor response assessment during the treatment interruption showed SD. The patient received two more curcumin infusions before the study was terminated due to disease progression. During the first 4 weeks of treatment, the investigator reported an improved general condition (WHO 2 to WHO 1) and a temporary reduction of LDH was observed from 435 to 202 U/L (normal range 135–225 U/L). In Patient #30 with colon cancer and liver and lung metastases, the CEA level was reduced from 18,542 µg/L at screening to 6441 µg/L, and CA19-9 was reduced from 18,105 U/mL at screening to 13,238 U/mL after 8 weeks (Fig. 3b), while tumor staging revealed PD. The patient received three more infusions due to clinical benefit and tumor marker responses. During this time, the tumor markers increased again and treatment was stopped after the 11th infusion due to a deterioration in general health. The patient died 13 days after last administration of the study drug. Both patients with tumor marker response were treated at a curcumin dose of 300 mg/m^2^ (Patient #27 over 8 h and Patient #30 over 6 h).

## Discussion

Patients participating in this study generally had advanced cancer, had exhausted lines of established anticancer treatments and were considered ineligible for later-phase clinical studies. Dose escalation of liposomal curcumin was continued until DL 6a (300 mg/m^2^ over 6 h). Infusions of liposomal curcumin over 8 h at doses between 100 and 300 mg/m^2^ (DL 1–6) were generally well tolerated, and the number of reported AEs and SAEs was not unexpected for the heavily pretreated patient population.

At DL 6a (300 mg/m^2^ over 6 h), the number of observed AEs related to the study drug increased significantly. At this DL, a significant decrease of Hb during infusions was observed in four of six patients. Definite hemolysis, meeting the definition of a DLT, was observed in one patient (#28), three other patients experienced anemia (#29, #31, #32). While the decrease of Hb observed in each of these patients was the reason for stopping dose escalation, comparison of changes in Hb in different patients should be interpreted with caution. These patients had different treatment histories and different degrees of prior bone marrow destruction secondary to different prior chemotherapies and in some patients, prior radiation therapy. All patients received multiple concomitant medications, some of which might have predisposed the red cells to hemolysis from curcumin. Four of the most common causes of drug-induced hemolysis are use of levofloxacin, cephalosporins, penicillins or ibuprofen [25–28]. Patient #28 was receiving levofloxacin (tavanic), amoxicillin and ibuprofen (brufen). Patient #32 was receiving a cephalosporin (zinnat) and had been receiving both a penicillin and ibuprofen shortly before starting liposomal curcumin. It was considered that there might be a threshold for Hb at the start of infusion below which the drop in Hb becomes more considerable. Based on the data from the previous phase 1a study [23, 29], echinocyte formation was expected, especially at higher DLs, with AUC and *C*_max_ comparable to phase 1a. However, echinocytes were observed in only one patient (#2), at DL 1 (100 mg/m^2^). In a heavily pretreated patient population, the frequency of adverse events is expected to increase with higher dose levels. While adverse events might be controlled in the hospital setting, this might not be possible in an external setting. For this reason, the dose of 300 mg/m^2^ liposomal curcumin monotherapy over 6 h is warranted as the recommended starting dose for future anti-cancer clinical trials.

PK analysis showed stable plasma concentrations of total curcumin and THC during infusion and a rapid decline after the end of infusion as a consequence of redistribution and extensive metabolism. It is well known that both curcumin and THC undergo a high degree of glucuronidation (phase 2 metabolism) following oral administration resulting in inactive metabolites [30]. We chose to focus the PK analysis on total plasma levels of curcumin (which would also include curcumin resulting from in vivo deconjugation) as it possesses anticancer activity. In addition, we chose to follow its phase 1 metabolism to the major metabolite THC which also possesses biological activity [31] by monitoring the total plasma levels of THC. At DL 300 mg/m^2^, the mean *C*_max_ plasma levels of curcumin were similar ranging between 1428 and 1641 ng/mL for the 6 and 8 h infusions. While the plasma levels of curcumin and THC of most patients dropped to unquantifiable levels within 10 min after EOI, there was one remarkable exception in Patient #19 whose curcumin plasma concentration was 251 ng/mL 45 min after EOI. The *C*_max_ and AUC of curcumin in this patient were also remarkably high and comparable to values observed in Patient #24. Analysis of possible factors that might influence the PK of curcumin is currently ongoing. Interestingly, the high curcumin plasma concentrations in patients #3, #19 and #24 did not result in the type of adverse events or the reduction of Hb that were reported for patients in DL 6a. In the remaining 29 patients, an apparent linear increase of plasma concentration with infusion rate was observed. However, for a fourfold change at higher infusion rates (12.5–50 mg/m^2^/h), there was a 24.0-fold change in the mean 2-h plasma levels of curcumin, suggesting that with increasing infusion rate, there were deviations from dose proportionality. This is even more apparent if one compares the *C*_max_ and AUC_0−Tlast_ values for doses of curcumin ranging from 190 to 300 mg/m^2^. Thus, at higher infusion rates of curcumin, even with robust plasma elimination mechanisms in place, the elimination of curcumin during infusion may be reaching saturation. In contrast to the finding of high levels of curcumin in patients #3, #19 and #24, the plasma levels and pharmacokinetics of curcumin and THC for the remaining patients were clearly dose-dependent and displayed a moderate to high, but not excessive amount of variability for such a diverse patient population.

Evaluation of antitumor activity was only a secondary study objective and tumor response according to RECIST v1.1 after 8 weeks was not expected in this heavily pretreated patient population, especially at low doses. Reductions of relevant tumor markers were observed in Patient #27 with prostate carcinoma and bone and lymph node metastases and lymphangiosis of the lung and in Patient #30 with colon carcinoma and liver and lung metastases. These were considered as objective signs of efficacy. Additionally, transient clinical benefit was reported by the investigator in both patients. Previous treatments for Patient #27 were radiation therapy and six prior chemotherapy combinations. Patient #30 had received seven prior chemotherapy combinations. Interestingly, the Hb in these two patients was rather constant compared to other patients at this dose level, who showed a greater reduction of Hb during the infusions.

In general, patients recruited into this early phase I study had failed previous anti-cancer treatments and often exhibited an aggressive course of disease. It might be that curcumin’s ability to kill cancer stem cells did not translate into tumor shrinkage due to the limited times of treatment and the impairment of immunological function in these patients. It is possible that the ideal role of liposomal curcumin as an anti-cancer agent should be in combination with other chemotherapies. The activity of curcumin as a sphingosine kinase inhibitor also suggests it may help in reducing the chances of recurrence in patients who have responded to other anti-neoplastic agents [32].

Supplementary information is available at the website of *Cancer Chemotherapy and Pharmacology*.
